# Prevalence and drivers of human scabies among children and adolescents living and studying in Cameroonian boarding schools

**DOI:** 10.1186/s13071-016-1690-3

**Published:** 2016-07-19

**Authors:** Emmanuel Armand Kouotou, Jobert Richie N. Nansseu, Michèle K. Kouawa, Anne-Cécile Zoung-Kanyi Bissek

**Affiliations:** Biyem-Assi District Hospital, Yaoundé, Cameroon; Faculty of Medicine and Biomedical Sciences, University of Yaoundé I, Yaoundé, Cameroon; Yaoundé General Hospital, Yaoundé, Cameroon; Sickle Cell Disease Unit, Mother and Child Centre, Chantal Biya Foundation, Yaoundé, Cameroon

**Keywords:** Epidemiology, Human scabies, School, Cameroon

## Abstract

**Background:**

The dire lack of information concerning the epidemiology of human scabies in Cameroon, especially in school milieus brought us to undertake the present study which aimed to determine the prevalence and associated factors of scabies in Cameroonian boarding schools.

**Methods:**

A cross-sectional study was conducted from February to March 2015 in four boarding schools in Yaoundé and Buea (Cameroon). Participants were students currently residing in one of the study sites, volunteering to participate in the study and whose parents or guardians had given their consent in this respect. The diagnosis was based on clinical assessment independently performed by two dermatologists.

**Results:**

A total of 1,902 students were recruited (50.5 % boys), with a mean age of 14.3 ± 2.5 years. Overall, 338 participants (17.8 %) were diagnosed with scabies. Age ≤ 15 years, male sex, number of students in the school > 500, no access to the school infirmary, sleeping with others, sharing beddings, clothes or toilet stuffs, pruritus in the close entourage and complaining of pruritus were significantly associated with the presence of mites in univariable logistic regression analyses. On the other hand, at least two baths per day, usage of soap for baths and finger nails always cut short appeared as protective factors. After multivariable analysis, male sex (adjusted OR (aOR) 2.06, 95 % CI: 1.40–3.01, *P* < 0.0001), first cycle level of education (aOR 1.67, 95 % CI: 1.02–2.71, *P* = 0.040), number of students per dormitory ≤ 10 (aOR 6.99, 95 % CI: 3.34–14.71, *P* < 0.0001), no access to the school infirmary (aOR 1.62, 95 % CI: 1.12–2.32, *P* = 0.009) and complaining of pruritus (aOR 93.37, 95 % CI: 60.04–145.19, *P* < 0.0001) were the independent factors associated with scabies.

**Conclusions:**

The prevalence of scabies was 17.8 %. The male sex, first cycle level of education, a number of students per dormitory ≤ 10, no access to the school infirmary and complaining of pruritus were the independent factors significantly impacting the occurrence of scabies.

## Background

Human scabies is an ectoparasitic infection of the skin caused by a mite, *Sarcoptes scabiei* var. *hominis*. It is ubiquitous, cosmopolitan, contagious and remains one of the most frequent skin diseases in resource-poor countries [1, 2]. The disease is transmitted through direct and prolonged contact with an infected skin, or rarely by using contaminated personal objects [1, 2]. It has a significant impact in terms of cost of treatment, absence at work or school and psychological repercussions [3, 4].

Scabies is a major public health problem in low-income areas given its burden and complications, especially in the paediatric population [1, 5]. In schoolchildren for instance, the infection often spreads quite rapidly, owing to their close contact and overcrowding within the schools [1, 2, 6]. Additionally, this health threat has been shown to negatively impact on learning [7]. Reports from a welfare home in Pulau Pinang (Malaysia), the Sivas orphanage (Turkey) and from Thai orphanages (Thailand) yielded a scabies prevalence of 31, 33 and 87.3 %, respectively [8–10]. In an Islamic religious school (Bangladesh), Talukder et al. [11] observed prevalences of 61–62 %. Besides, in a study including 944 students of secondary boarding schools in Kuching (Sarawak, Malaysia), Yap et al. found 8.1 % cases of human scabies [11].

In Africa, studies conducted in school milieus revealed a prevalence of scabies infestation of 4.4, 10.5, 4, 0.7 and 8.3 % in Egypt, Nigeria, Mali, Malawi and Kenya, respectively [6, 7, 12, 13]. However, there is a crucial lack of data regarding the burden of human scabies in school milieus in Cameroon, a sub-Saharan African country. To fill this gap, we undertook the present study, aiming to determine the prevalence and risk factors for human scabies in boarding schools in Cameroon.

## Methods

### Ethical statement

Prior to commencing this study, institutional authorisations were obtained from the authorities in charge of Secondary Education and from the administrative authorities of each study site. Besides, an ethical clearance was issued by the Ethical Review Board of the Faculty of Medicine and Biomedical Sciences of the University of Yaoundé I, Cameroon. Information leaflets and consent forms were sent to each student’s parent or guardian, presenting all the aspects and procedures of the study, and we enrolled only students whose parents/guardians returned a signed consent form, hence authorising the investigators to include the child in the study. The present study did not interfere with the lessons at the study site, and the students who did not participate were neither reprimanded nor sanctioned.

### Study design, setting and participants

From February to March 2015, we conducted a cross-sectional study in four boarding schools in Cameroon. Of these, three were randomly chosen in Yaoundé (Centre Region of the country), namely the Pi and Ju (PNU) College, the Baptist High School (BHS) and the Christian Comprehensive Secondary School (CCSS). The fourth school, the Presbyterian Comprehensive Secondary School (PCSS), was randomly chosen among the boarding schools located in Buea (South-West region of Cameroon). Yaoundé and Buea were conveniently chosen, influenced by some facilities the investigators had in these towns, the study receiving neither funding nor sponsorship.

Participants were students, regardless of their level of education, currently residing in one of the study sites, volunteering to participate in the study and whose parents or guardians had given their consent in this respect. They were consecutively and exhaustively recruited during the study period. Students absent from the school when the investigators visited, or those refusing to participate were not included.

### Data collection

Before recruitment, sensitisation sessions were organized by the investigators in each study site to inform the authorities and students of the various aspects of the study, emphasising on the huge advantages of taking part in it. Recruitments were performed at distance from lessons, in an adapted space prepared the day before, by a team of dermatologists, medical students and nurses.

Data were collected during an interview conducted by an investigator using a standardised and pretested questionnaire. Data collection comprised socio-demographic characteristics (age, sex, class attended and family size), history of pruritus (witnessed by the student or his/her entourage and the prevailing period) and frequency of baths and laundries.

Subsequently, a physical examination was independently undertaken by two experienced dermatologists. The diagnosis was ascertained, based on a clinical algorithm which has yielded a sensitivity of 100 % and a specificity of 96.9 % [14]. Accordingly, a student complaining of pruritus, on whom scabies lesions were notified at least at two specific body sites with or without history of pruritus in the close entourage was declared suffering from scabies. Dermoscopy and/or skin scrapings/microscopy were not available to confirm the diagnosis. Disagreements between the dermatologists were reconciled through discussion and consensus. Students found infected received anti-scabies medications made of benzyl benzoate along with their entourage, given free of charge. Additionally, the bedding and laundry had to be disinfected.

### Statistical methods

Data were analysed using SPSS version 20.0 (IBM, Chicago, Illinois, USA). Results are presented as frequency (percentage) or mean ± standard deviation (SD) where appropriate. The Chi-square test or Fisher’s exact test served for the comparison of qualitative variables. Student’s *t-*test or equivalents were used for the comparison of quantitative variables. Odds ratios with 95 % confidence intervals (CI) served to assess the factors impacting the presence of scabies; they were calculated with univariable and multivariable regression analyses while adjusting for confounding factors. All variables with *P* < 0.25 in univariable analyses (and pertinent variables) were introduced in the multivariable model. The level of statistical significance was set at *P*-value < 0.05.

## Results

Of the 2,235 students regularly registered in the four study sites, 152 were absent when the investigators visited and 181 refused to take part in the study. On the whole, 1,902 students were enrolled in this study, hence a participation of 85.1 %. Ages ranged from 9 to 22 years, with a mean of 14.3 ± 2.5 years. The most represented age group was 11–15 years (65.8 %), followed by the group 16–20 years (30.8 %). There were 960 boys (50.5 %), hence a M/F sex ratio of 1.02/1. We had 1,254 students (65.9 %) from the first cycle and 648 (34.1 %) from the second one. A total of 413 students (21.7 %) complained of pruritus, mainly at night and leading to insomnias among 278 students (67.3 %).

We diagnosed 338 participants (17.8 %) with human scabies among whom 223 boys (66.0 %). There were significantly more infected boys than girls (23.3 % *vs* 12.2 %; *χ*^2^ = 39.521, *df* = 1, *P* < 0.0001; Table 1). Ages of these infected students ranged between 9 and 20 years, with a mean of 13.5 ± 2.3 years. Students with scabies were younger than those without (mean 13.5 *vs* 14.5 years; *t*-test, *t* = 6.585, *df* = 1900, *P* < 0.0001); they were from boarding schools with significantly higher number of registered students (mean 914.3 *vs* 732.7 students; *t*-test, *t* = 9.554, *df* = 571.069, *P* < 0.0001). The number of students staying in the same dormitory was lower among students with scabies compared to those without (mean 25.8 *vs* 32.0 students; t- test, *t* =7.191, *df* = 571.069, *P* < 0.0001), as well as the number of students sharing the same toilet (mean 52.3 *vs* 63.1 students; *t*-test, *t* = 2.122, *df* = 109.382, *P* = 0.036).

**Table 1:** Comparison between students with and without scabies, and univariable and multivariable logistic regression analyses

Variable	Without scabies (*n* = 1,564)	With scabies (*n* = 338)	OR (95 % CI)	*P*-value	aOR (95 % CI)^a^	*P-*value
	Number (%)	Number (%)				
Age group (years)						
≤ 15	1,039 (79.6)	267 (20.4)	1.90 (1.43–2.52)	< 0.0001^*^	1.11 (0.65–0.87)	0.708
> 15	525 (88.1)	71 (11.9)	1		1	
Sex						
Females	827 (87.8)	115 (12.2)	1		1	
Males	737 (76.8)	223 (23.2)	2.18 (1.70–2.78)	< 0.0001^*^	2.06 (1.40–3.01)	< 0.0001^*^
Level of education						
First cycle	1,027 (81.9)	227 (18.1)	1.07 (0.83–1.37)	0.599	1.67 (1.02–2.71)	0.040^*^
Second cycle	537 (82.9)	111 (17.1)	1		1	
Number of students in the school						
≤ 500	438 (90.9)	44 (9.1)	1		1	
> 500	1,126 (79.3)	294 (20.7)	2.60 (1.86–3.64)	< 0.0001^*^	1.65 (0.99–2.74)	0.054
Number of students using the same toilet						
≤ 10	25 (86.2)	4 (13.8)	1			
> 10	1,539 (82.2)	334 (17.8)	1.36 (0.47–3.92)	0.574	/	/
Number of students per dormitory						
≤ 10	84 (77.8)	24 (22.2)	1.35 (0.86–2.16)	0.214	6.99 (3.34–14.71)	< 0.0001^*^
> 10	1,480 (82.5)	314 (17.5)	1		1	
Number of baths						
At least twice daily	73 (61.3)	46 (38.7)	0.31 (0.21–0.46)	< 0.0001^*^	0.52 (0.25–1.07)	0.076
Less than twice daily	1,491 (83.6)	292 (16.4)	1		1	
Access to infirmary						
No	772 (80.3)	189 (19.7)	1.30 (1.03–1.65)	0.029^*^	1.62 (1.12–2.32)	0.009^*^
Yes	792 (84.2)	149 (15.8)	1			
Sleeping with others						
No	1,494 (83.6)	293 (16.4)	1		1	
Yes	70 (60.9)	45 (39.1)	3.28 (2.21–4.87)	< 0.0001^*^	1.29 (0.69–2.41)	0.433
Sharing of beddings, clothes or toilet stuffs						
No	677 (85.4)	116 (14.6)	1		1	
Yes	887 (80.0)	222 (20.0)	1.46 (1.14–1.87)	0.002^*^	1.08 (0.73–1.59)	0.700
Usage of soap for baths						
No	150 (65.2)	80 (34.8)	1		1	
Yes	1,414 (84.6)	258 (15.4)	0.34 (0.25–0.46)	< 0.0001^*^	0.76 (0.44–1.32)	0.329
Finger nails always cut short						
No	352 (74.1)	123 (25.9)	1		1	
Yes	1,212 (84.9)	215 (15.1)	0.51 (0.40–0.65)	< 0.0001^*^	0.84 (0.55–1.30)	0.439
Ironing of clothes and bedding						
No	1,220 (81.8)	272 (18.2)	1			
Yes	344 (83.9)	66 (16.1)	0.86 (0.64–1.16)	0.317	/	/
Pruritus in the close entourage						
No	999 (90.2)	108 (9.8)	1		1	
Yes	565 (71.1)	230 (28.9)	3.77 (2.94–4.84)	< 0.0001^*^	1.30 (0.87–1.93)	0.196
Complaining of pruritus						
No	1,441 (96.8)	48 (3.2)	1		1	
Yes	123 (29.8)	290 (70.2)	70.78 (49.55–101.11)	< 0.0001^*^	93.37 (60.04–145.19)	< 0.0001^*^

*Abbreviations*: *aOR* adjusted odds ratio, *CI* confidence interval, *OR* odds ratio

^a^All variables with *P* < 0.25 in univariable analyses (plus level of education) were introduced in the multivariable model. The coefficient of determination of this model was *R* ^*2*^ of McFadden = 0.520 (*P* < 0.0001)

^*^ *P*-value < 0.05

Table 1 displays the univariable and multivariable regression analyses. In univariable analyses, age ≤ 15 years, male sex, number of students in the school > 500, no access to the school infirmary, sleeping with others, sharing beddings, clothes or toilet stuffs, pruritus in the close entourage and complaining of pruritus were significantly associated with the presence of mites. On the other hand, at least two baths per day, usage of soap for baths and finger nails always cut short appeared as protective factors (Table 1). After multivariable analysis, male sex (adjusted OR (aOR) 2.06, 95 % CI: 1.40–3.01; *P* < 0.0001), first cycle level of education (aOR 1.67, 95 % CI: 1.02–2.71; *P* = 0.040), number of students per dormitory ≤ 10 (aOR 6.99, 95 % CI: 3.34–14.71; *P* < 0.0001), no access to the school infirmary (aOR 1.62, 95 % CI: 1.12–2.32; *P* = 0.009) and complaining of pruritus (aOR 93.37, 95 % CI: 60.04–145.19; *P* < 0.0001) were the independent factors associated with scabies.

Concerning the sites of lesions, interdigital spaces (61.2 %), wrists (54.4 %), buttocks (48.5 %) and elbows (39.1 %) were the main sites (Table 2). Lesions were diffused in 13.0 % of cases. Moreover, papules (75.4 %) and scratch marks (66.3 %) were the most recorded non-specific lesions whereas pearled vesicles (43.2 %) and scabious nodules (33.1 %) were the prevailing specific lesions of the disease (Table 2).

**Table 2:** Sites and types of lesions

Characteristic		Number (%)
Type of lesions		
Non-specific lesions	Hyperkeratosic and crusty lesions	129 (38.2)
	Papules	255 (75.4)
	Pustules	26 (7.7)
	Scratch marks	224 (66.3)
Specific lesions	Pearled vesicles	146 (43.2)
	Scabious nodules	112 (33.1)
Site of lesions	Foot	54 (16.0)
	Axillary creases	83 (24.6)
	Interdigital spaces	207 (61.2)
	Wrists	184 (54.4)
	Elbows	132 (39.1)
	Buttocks	164 (48.5)
	Anterior face of thighs	109 (32.3)
	Glans	31 (9.2)
	Breasts	9 (2.7)
	Anterior armholes	116 (34.3)
	Posterior armholes	90 (26.6)
	Inguinal creases	85 (25.2)
	Diffused	44 (13.0)

## Discussion

This study conducted in four boarding schools in Cameroon identified 17.8 % cases of human scabies. The male sex, first cycle level of education, a number of students per dormitory ≤ 10, no access to the school infirmary and complaining of pruritus were the independent factors significantly impacting the occurrence of scabies. Sites and types of infection resembled what was observed in a previous study [15].

The prevalence of scabies in this study (17.8 %) is higher than what has been reported from Malaysian secondary boarding schools (8.1 %) and some African school milieus, between 0.7 and 13 % [6, 7, 12, 16]. It is comparable to the 18.5 % found by Pasay et al. [17], but is lower than what was witnessed among children in a Malaysian welfare home (31 %), in a Turkish orphanage (33 %), in a Bangladesh Islamic religious school (61–62 %), in a Sierra Leone displacement camp (67 %) and in Thailand orphanages (87.5 %) [8–10, 18, 19]. Likewise, high rates of scabies infestation have been observed in prisons, around 41–57 % [20, 21]. High rates of scabies are usually found in communities and milieus where overcrowding and poverty are highly prevalent [1, 2], perhaps explaining the discrepancies between our results and other ones.

Indeed, although the number of students in the school > 500 was a significant risk factor in univariable analysis, it became nonsignificant in the multivariable model, may be inferring that these schools where not too congested to lead to the spreading of the infection. It is true however that the result in the multivariable model could have been different with a different cut-off point; the 500-student cut-off was chosen arbitrarily. A number of students per dormitory ≤ 10 was also associated with scabies, a paradoxical and troubling finding as we would have expected the contrary. Nonetheless, we observed that these students were more prone to bathe less than twice daily compared to their counterparts (*P* = 0.019), showing perhaps that they had a poorer personal hygiene which can therefore explain the results.

Being younger (≤ 15 years old) was also a risk factor in univariable analysis but not in the multivariable one, compared with first cycle level of education which remained significant in the multivariable model. Considering that the spreading of human scabies is linked with poor personal hygiene [1, 2], it is possible that the younger students, who are of lower educational level than their counterparts, are less aware of personal hygiene rules to adopt especially when living with others, being therefore more prone to be infected. We observed in fact that younger students were significantly more prone to share their beddings or clothes with others, sleep with others and have a history of pruritus in their close entourage than the elders (all *P*-values < 0.0001).

The male sex was a risk factor for scabies infestation, a finding differing from Hegab et al’s report in Egypt where there was no difference between males and females [6]. We noticed that more boys used to sleep with others (*P* = 0.001), more had a history of pruritus in their close entourage (*P* = 0.006), less used soap for bathing (*P* < 0.0001) and less had their finger nails always cut short (*P* < 0.0001) than girls. These observations may explain why the male sex was a risk factor for scabies in this study. Sleeping with others had already been identified as a risk factor for scabies infestation [6, 22], as well as sharing of clothes with others [6]. However, these variables which were significant in univariable analyses became nonsignificant in our multivariable model.

Lack of accessibility to the school infirmary was independently linked with scabies (aOR 1.62). In fact, considering the high contagiousness of human scabies which is transmitted by contact with an infected skin [1, 2], it is clear that if a student is infected and has no access to the infirmary to consult and be treated, he/she will be a continuous point of spreading of the disease. Moreover and unsurprisingly, students complaining of pruritus had a 93.4-fold increased risk of being diagnosed with scabies, given the highly itching character of this skin infection. The itching can become very intense and uncomfortable, so much so that it may affect the quality of life of the individuals affected [4]. We noticed in this concern that a significant number of infected students complaining of pruritus were experiencing insomnias in comparison to their counterparts without scabies (*P* < 0.0001). This may negatively impact the school performances of the student [7]. We need further studies to thoroughly investigate the quality of life and negative effects of scabies in students and in the general population.

Some limitations of our study are to be mentioned. First, dermoscopy and/or skin scrapings/microscopy were not available to confirm the presence of mites, although they are operator-dependent and have relatively low sensitivities around 90–91 % [23, 24]. Our diagnosis was essentially based on clinical assessment independently conducted by two dermatologists, on the basis that clinical assessment of human scabies has been shown with good sensitivity and specificity: 100 and 96.9 %, respectively [14]. Secondly, the cross-sectional design of this study precludes us from affirming with conviction that the predictors identified are really independent risk factors for scabies in these boarding schools. Thirdly, our findings might not be generalised to the entire population of Cameroon boarding students given that we worked only in four schools; however, these study sites were randomly chosen in the Centre and South-West regions of the country.

## Conclusion

We found from this study of 1,902 students recruited in four boarding schools in Cameroon that the prevalence of human scabies was 17.8 %. The male sex, first cycle level of education, a number of students per dormitory ≤ 10, no access to the school infirmary and complaining of pruritus were the independent factors significantly impacting the occurrence of scabies. Strong measures should be taken to ameliorate accessibility of students to school infirmaries and provide these facilities with enough resources to take care of children health issues at school. Additionally, personal hygiene rules should be taught to students, with special emphasis among boys and the younger students. More studies to better elucidate all the factors driving the occurrence of scabies and its impact on quality of life of the children affectedare warranted in our contexts.

## Abbreviations

aOR, adjusted odds ratio; OR, odds ratio; SD, standard deviation
